# Rice body synovitis in children: a retrospective study of 6 cases and a systematic literature review of the last two decades (2006–2026)

**DOI:** 10.3389/fped.2026.1801834

**Published:** 2026-05-13

**Authors:** Qiang Ren, Jing Feng, Xuyang Cao, Jingyan Li, Tuokang Zheng, Yanhua Feng

**Affiliations:** 1 Department of Emergency Surgery, Children’s Hospital of Hebei Province; Hebei Provincial Clinical Research Center for Child Health and Disease, Shijiazhuang, Hebei, China; 2 Department of Orthopaedics, North China Medical Health Group Xingtai General Hospital, Xingtai, Hebei, China; 3 Department of Emergency, The Second Hospital of Hebei Medical University, Shijiazhuang, Hebei, China

**Keywords:** arthroscopic surgery, juvenile idiopathic arthritis, pediatric orthopedics, rice body synovitis, systematic review

## Abstract

**Objective:**

Rice body synovitis (RBS), historically linked to tuberculosis, is increasingly recognized as an articular manifestation of autoimmune diseases in children, particularly Juvenile Idiopathic Arthritis (JIA). However, its diagnosis remains challenging and its management often fragmented between rheumatology and orthopedics. This study aims to characterize RBS within the spectrum of pediatric rheumatic disease and propose an integrated management framework.

**Methods:**

We conducted a retrospective analysis of six pediatric patients (aged 2–9 years) with knee RBS, all of whom underwent arthroscopic debridement. Concurrently, a systematic literature review (2006–2026) was performed to synthesize global evidence on etiology, diagnosis, and treatment. Descriptive statistics were utilized to analyze the pooled cohort.

**Results:**

In our cohort, RBS was associated with JIA (*n* = 1), tuberculosis (*n* = 1), and non-specific synovitis (*n* = 4). Magnetic resonance imaging (MRI) demonstrated high diagnostic specificity, showing characteristic hypointense “rice bodies” within hyperintense effusion on T2-weighted sequences. Surgical debridement provided immediate symptom relief, but recurrence occurred in the JIA patient, necessitating a combination of standard and biologic disease-modifying antirheumatic therapy (DMARD). Integrating literature data yielded a pooled cohort of 44 patients, confirming JIA as the predominant etiology (77.3%) in contemporary practice, highlighting an emerging trend moving away from strictly infectious to autoimmune origins.

**Conclusions:**

RBS represents a rare but clinically significant articular phenotype within pediatric rheumatology, most commonly linked to JIA. Successful management requires a dual strategy: (1) MRI-guided early diagnosis and (2) a collaborative rheumatology-orthopedics approach combining arthroscopic intervention with targeted immunomodulatory therapy. Critically, the interpretation of etiology must be contextualized by geographical variability; while JIA predominates in developed nations, mycobacterial infections remain a paramount diagnostic priority in tuberculosis-endemic regions.

## Introduction

Rice body synovitis (RBS) represents a unique and relatively rare pathological response of the synovial membrane, characterized by the formation of countless loose, polished, white, or off-white granules within the joint space, bursae, or tendon sheaths (1). Historically, this condition was first described by Riese in 1,895 in the context of tuberculous arthritis, leading to a long-standing clinical axiom associating “rice bodies” with mycobacterial infection (2). However, the landscape of this disease has evolved significantly over the last century. Contemporary literature suggests that RBS is a non-specific endpoint of chronic synovial inflammation, increasingly identified in association with systemic autoimmune disorders such as Rheumatoid Arthritis (RA), Juvenile Idiopathic Arthritis (JIA), and seronegative spondyloarthropathies, rather than infection alone (3, 4).

The pathophysiology underlying rice body formation remains a subject of debate, with two primary hypotheses dominating the academic discourse. The “synovial microinfarction theory” posits that chronic hypertrophy of the synovial villi leads to distal ischemia and microinfarction. These necrotic villi subsequently detach into the joint cavity and are encased in layers of fibrin (5). Conversely, the “*de novo* fibrin aggregation theory” suggests that activated Type B synoviocytes secrete excessive fibrinogen and collagen, which aggregate directly in the inflammatory synovial fluid to form the core of these bodies, independent of synovial shedding (6, 7). Regardless of the mechanism, the presence of rice bodies indicates a severe, chronic inflammatory environment that requires aggressive intervention.

In the pediatric population, RBS presents a formidable diagnostic challenge. Unlike adults, children may present with atypical symptoms, and the condition is frequently misdiagnosed as synovial chondromatosis, Pigmented Villonodular Synovitis (PVNS), or culture-negative septic arthritis (8). Misdiagnosis can lead to delayed treatment, joint destruction, and growth disturbances. Furthermore, distinguishing between infectious (tuberculous or fungal) and autoimmune (JIA) etiologies is critical, as the therapeutic algorithms are diametrically opposed—immunosuppression for JIA could be catastrophic for an occult infection (9).

Despite its clinical significance, reports on pediatric RBS are scarce and predominantly limited to isolated case reports. There is a lack of consolidated data regarding the distribution of etiologies, the efficacy of specific imaging signs (such as the MRI “bullseye sign”), and long-term recurrence rates in children. To address this gap, we conducted a retrospective study of six pediatric cases treated at our institution. Furthermore, to provide a higher level of evidence and contextualize our findings, we performed a systematic literature review covering the period from 2006 to 2026. This combined approach aims to update the understanding of pediatric RBS, emphasizing the emerging trend in etiological spectrum and the necessity for a multidisciplinary treatment approach involving both orthopedics and rheumatology.

## Methods

### Study design and ethical considerations

This study was conducted in accordance with the Declaration of Helsinki and reported following the PROCESS guidelines for case series (10). The research protocol was approved by the Ethics Committee of the Children's Hospital of Hebei Province (No. 2025009). Informed consent was obtained from the parents or legal guardians of all participating patients for the publication of clinical data and accompanying images.

### Systematic literature review strategy

**Figure 1:**
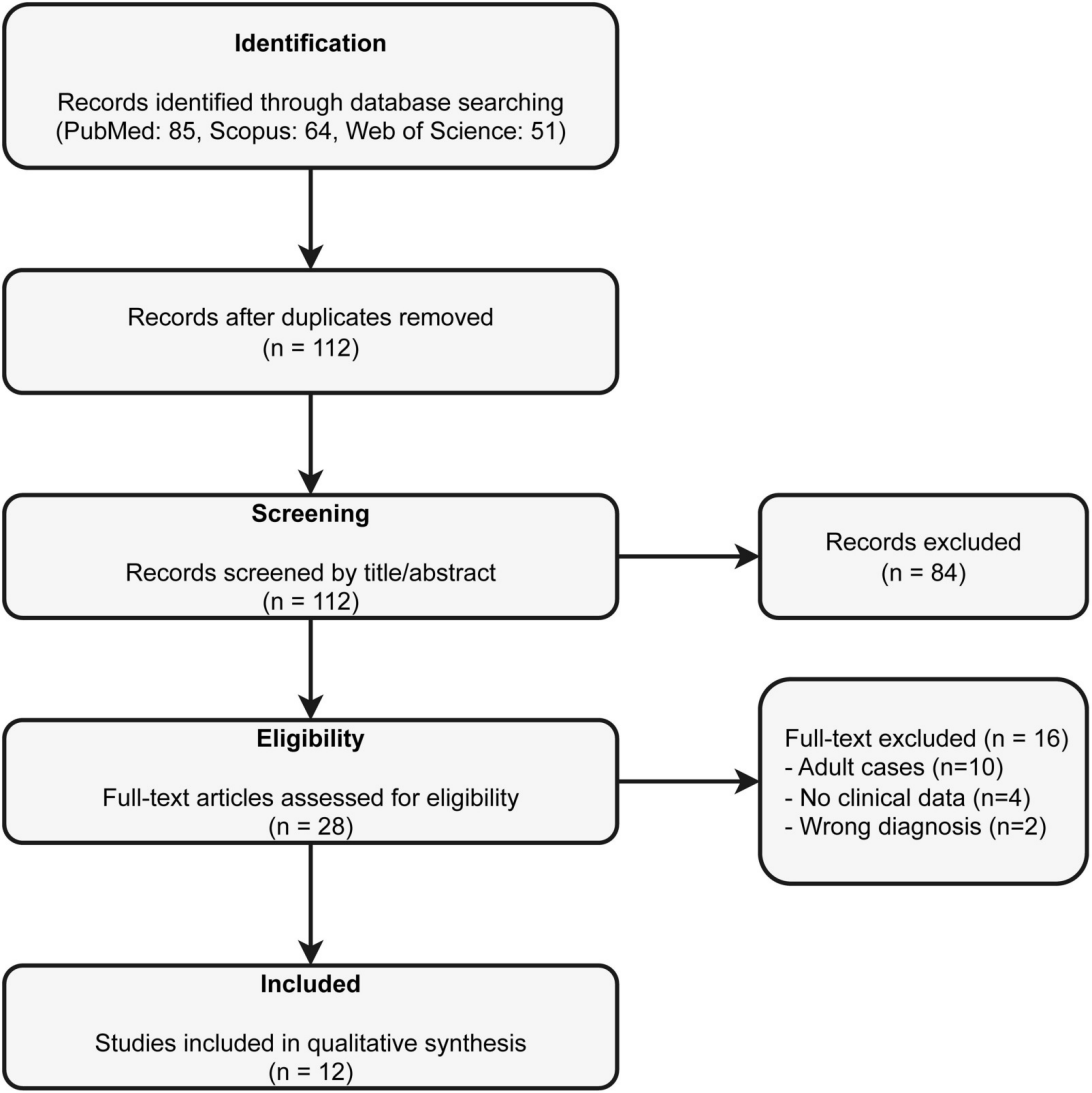
PRISMA flow diagram detailing the study selection process for the systematic literature review (2006–2026).

To contextualize our findings within the global landscape of pediatric RBS, a systematic literature review (SLR) was performed following the PRISMA (Preferred Reporting Items for Systematic Reviews and Meta-Analyses) guidelines (11). We searched PubMed, Web of Science, and Scopus databases for the period from January 1, 2006, to January 1, 2026. The exact search string used for PubMed was: ((“Rice body”[Title/Abstract] OR “Rice bodies”[Title/Abstract] OR “Rice body synovitis”[Title/Abstract]) AND (“Child”[Title/Abstract] OR “Pediatric”[Title/Abstract] OR “Adolescent”[Title/Abstract] OR “Juvenile”[Title/Abstract])). Similar Boolean operators were adapted for Web of Science and Scopus. Although this review was not registered with PROSPERO, at the time of review conceptualization, registration with PROSPERO was not feasible as data extraction and synthesis had already commenced, which precludes registration according to their current guidelines. Study screening and data extraction were performed independently by two authors (QR and JF), with discrepancies resolved by a third reviewer (YF). Quality control and risk-of-bias assessments were conducted using the Joanna Briggs Institute (JBI) critical appraisal tools for case reports and case series. A PRISMA flow diagram detailing the study selection process is provided in Figure 1. Inclusion criteria were: (1) patients aged 0–18 years; (2) confirmed diagnosis of RBS via MRI or pathology; and (3) availability of detailed clinical data. Exclusion criteria included adult cases and synovial chondromatosis without fibrin rice bodies. Ultimately, 12 relevant studies were identified for analysis.

### Patient selection and clinical evaluation

We retrospectively included six pediatric patients diagnosed with RBS of the knee joint admitted to our institution between June 2017 and October 2023. The detailed demographic and clinical characteristics are presented in Table 1. All patients underwent a comprehensive preoperative workup, including physical examination, knee ultrasonography, plain radiography, and magnetic resonance imaging (MRI). Laboratory investigations included complete blood count, erythrocyte sedimentation rate (ESR), C-reactive protein (CRP), rheumatoid factor (RF), antinuclear antibody (ANA), and T-SPOT.TB assays to screen for underlying autoimmune or infectious etiologies.

**Table 1:** General information and clinical characteristics of the 6 pediatric patients with RBS.

**Case no.**	**Sex**	**Age (y)**	**Length of hospital stay**	**RF Status**	**ANA Status**	**TB Screening (T-SPOT)**	**Etiological Diagnosis**
1	Male	6	2 Months	−	−	+	Tuberculous Synovitis (Clinical)
2	Female	8	3 Weeks	−	−	−	Non-specific/Seronegative
3	Female	2	1 Week	+	+	−	JIA (Polyarticular, RF+, ANA+)
4	Female	7	6 Months	−	−	−	Non-specific/Seronegative
5	Male	6	4 Months	−	−	−	Non-specific/Seronegative
6	Female	9	3 Months	−	−	−	Non-specific/Seronegative

### Surgical procedure

All six patients underwent exploratory knee arthroscopy and debridement under general anesthesia. Standard anteromedial and anterolateral portals were established. Intraoperatively, the joint cavity was inspected for the extent of synovial hypertrophy and the presence of loose bodies. An orthopedic shaver was used to systematically remove the rice bodies and perform a subtotal synovectomy. Care was taken to preserve the menisci and cruciate ligaments. The resected specimens were sent for histopathological examination and microbial culture (including mycobacterial culture). To rigorously rule out tuberculosis, all patients underwent intraoperative synovial tissue and fluid sampling for acid-fast bacilli (AFB) staining, mycobacterial culture, and Xpert MTB/RIF (PCR) testing.

### Postoperative management and follow-up

Postoperatively, skin traction was applied for one week. Rehabilitation, including non-weight-bearing range of motion (ROM) exercises, commenced after one week. Specific pharmacological treatments were initiated based on the final diagnosis [e.g., anti-tuberculosis therapy for Case 1 or disease-modifying antirheumatic drugs (DMARDs) for Case 3]. Patients were followed up for a mean period of 18.5 ± 4.2 months (range: 12–24 months). Clinical outcomes were evaluated using the Lysholm knee score and joint circumference difference. Pain intensity was assessed utilizing age-appropriate tools: the Visual Analogue Scale (VAS, 0–10) was used for children aged ≥6 years, while the FLACC scale (Face, Legs, Activity, Cry, Consolability) was utilized for the 2-year-old patient (Case #3) and converted to a 0–10 metric for data consistency in Table 2.

**Table 2:** Comparison of preoperative and final follow-up clinical outcomes.

Case no.	Pre-op Pain (VAS/FLACC^a^)	Final Pain (VAS/FLACC^a^)	Activity Limit (Pre/Final)	Swelling Diff. (cm) (Pre/Final)	Lysholm Score (Pre/Final)
1	5	0	Yes/No	6.95/0.05	38/100
2	1	0	Yes/No	4.30/0.10	67/100
3	3 (FLACC)	0	Yes/No	5.40/1.31	72/90
4	7	0	Yes/No	5.11/0.16	29/100
5	6	0	Yes/No	6.11/0.32	40/100
6	7	0	Yes/No	7.89/0.10	32/100

a Pain Assessment: Visual Analogue Scale (VAS, 0−10) was used for children ≥6 years; FLACC scale (0−10) was utilized for the 2-year-old patient (Case 3) due to cognitive inability to use VAS.

### Statistical analysis

Statistical evaluation was performed from a descriptive perspective to synthesize findings from our institutional cohort and the systematic review. Categorical variables (e.g., etiology, treatment modality) were expressed as frequencies (n) and percentages (%). Clinical outcome scores were presented as pre- and post-intervention paired values. Given the rarity of the condition and the pooling of case reports, formal inferential statistical tests for significance were not performed to avoid over-interpretation of heterogenous data.

## Results

### Part I: case series findings

#### Clinical and imaging characteristics

**Figure 2:**
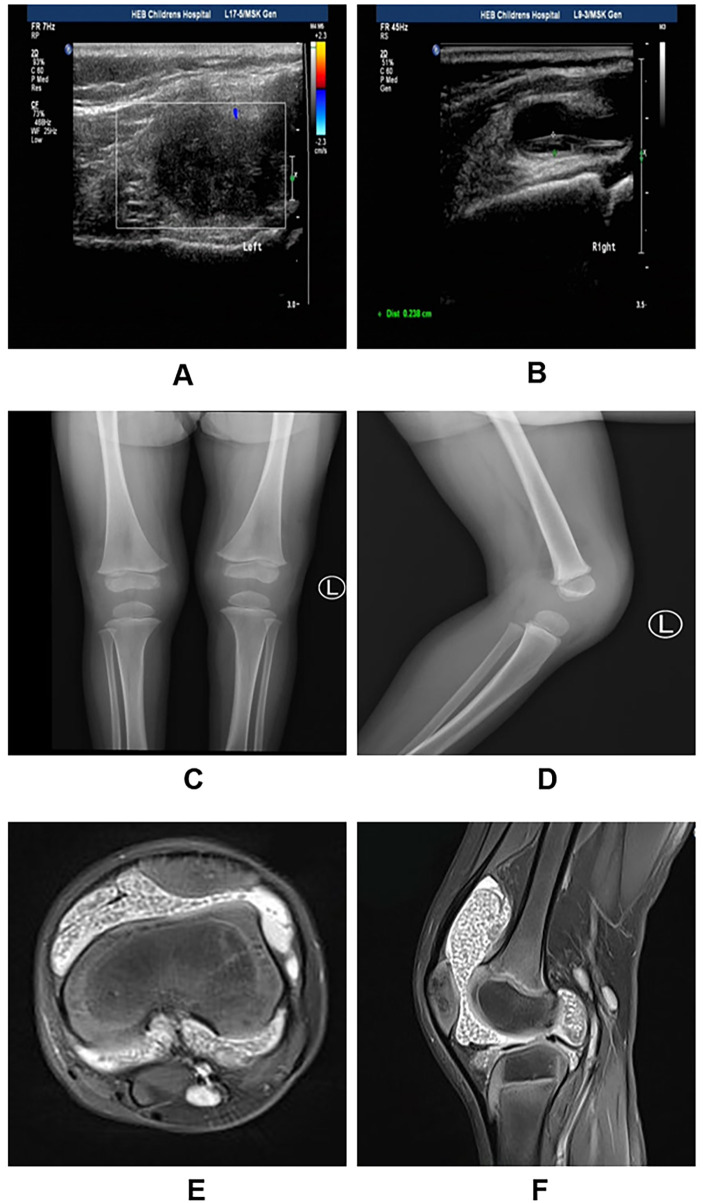
Multimodal preoperative imaging assessment of pediatric rice body synovitis. **(A,B)** Ultrasonography revealing marked synovial thickening with multiple hypoechoic nodules floating within the effusion. **(C,D)** Anteroposterior and lateral radiographs showing significant soft tissue swelling (distension of the suprapatellar pouch) without bone erosion. **(E,F)** Magnetic Resonance Imaging. **(E)** T2-weighted sagittal image demonstrates a massive joint effusion containing numerous hypointense, discrete nodules (white arrows). **(F)** T1-weighted axial image shows isointense nodules filling the joint capsule. Note the absence of blooming artifact, helping to distinguish from PVNS.

The study cohort consisted of 2 males and 4 females with an average age of 6.3 years. Preoperative imaging played a pivotal role in diagnosis (Figure 2). Ultrasonography consistently revealed synovial thickening with multiple hypoechoic nodules (Figures 2A,B). Radiographs showed soft tissue swelling but no osseous erosion (Figures 2C,D). MRI was diagnostic, demonstrating joint effusion and synovial proliferation containing numerous diffuse, circular signal intensities. These “rice bodies” appeared isointense on T1-weighted imaging (Figure 2F) and hypointense on T2-weighted imaging relative to the hyperintense synovial fluid (Figure 2E), exhibiting the characteristic “black pearl” or “bullseye” appearance.

#### Surgical and pathological findings

**Figure 3:**
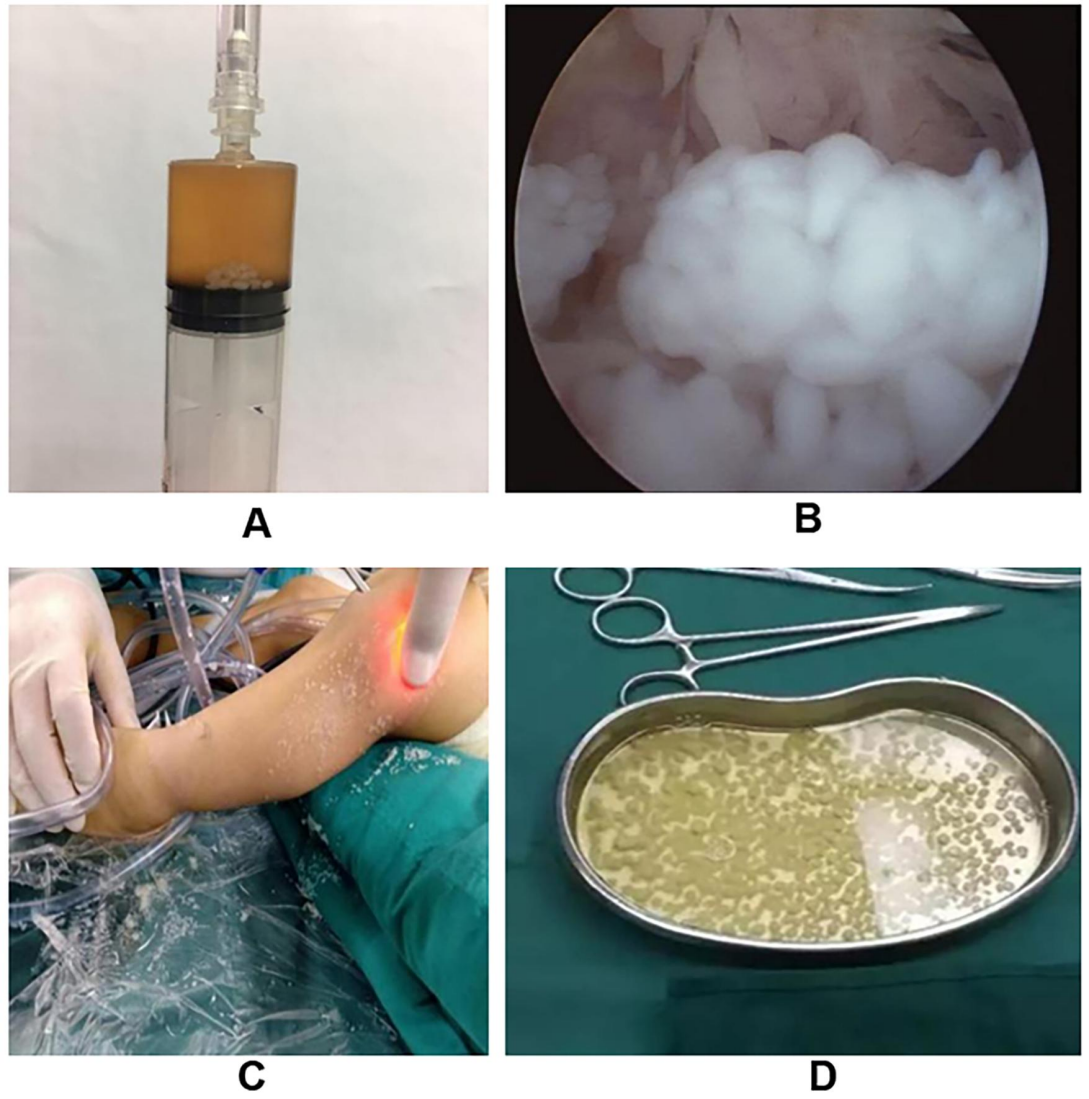
Intraoperative and macroscopic findings. **(A)** Joint aspiration yielding synovial fluid containing numerous rice-grain-sized fibrin bodies. **(B)** Arthroscopic view showing hyperemic, villous synovial proliferation filling the joint compartment. **(C)** Lavage demonstrating the outflow of large quantities of loose rice bodies. **(D)** Gross specimen of the resected rice bodies, showing their uniform, smooth, off-white, and granular nature.

**Figure 4:**
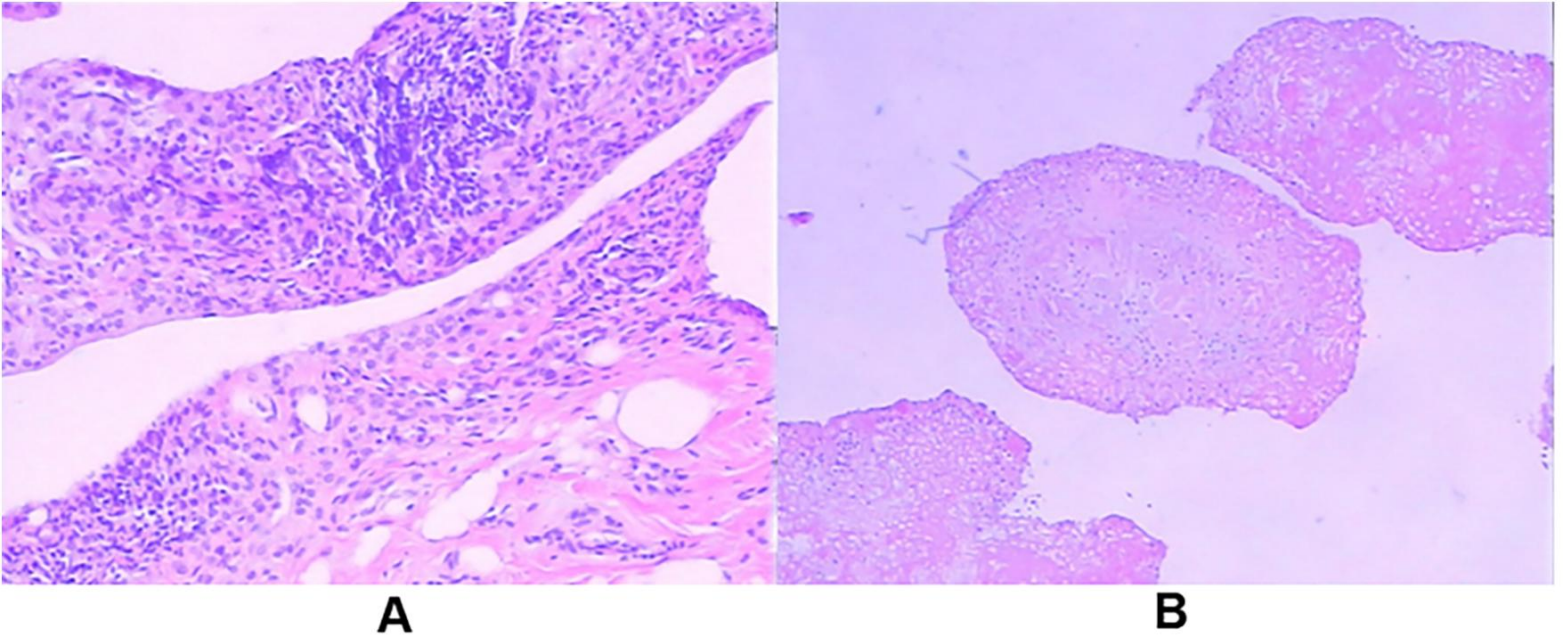
Histopathological analysis. **(A)** Low magnification (H&E, x100) showing synovial hyperplasia with dense lymphocytic infiltration. **(B)** High magnification (H&E, x400) of a rice body demonstrating an amorphous fibrin core (asterisk) surrounded by mononuclear cells. No caseating granulomas or malignant cells were observed. Scale bar = 100 μm.

Initial joint aspiration yielded synovial fluid containing numerous macroscopic, rice-grain-sized fibrin bodies (Figure 3A). Arthroscopic visualization confirmed the presence of hundreds of off-white, polished, grain-like particles (Figure 3C), either floating freely or loosely attached to the hyperemic synovial membrane (Figure 3B). Gross examination of the resected specimens showed uniform granular bodies (Figure 3D). Histopathological analysis (Figure 4A) revealed hyperplastic synovial villi with significant lymphocytic infiltration. The rice bodies consisted of an amorphous eosinophilic fibrin core surrounded by a thin layer of histiocytes or fibrin (Figure 4B), consistent with non-specific chronic inflammation. Notably, although typical caseating granulomas were absent in Case #1, the combination of positive T-SPOT results and chronic synovitis led to a clinical diagnosis of tuberculous synovitis. All other cases were confirmed negative for mycobacterial and bacterial infections via intraoperative PCR (Xpert MTB/RIF) and prolonged cultures.

#### Treatment outcomes and management of comorbidities

Surgical wounds healed without complications in all patients. Postoperative outcomes are summarized in Table 2. Pain scores decreased significantly, and Lysholm scores improved to 100 in 5 out of 6 patients.

Regarding specific etiological management:

- **Case #1 (TB Positive):** Given the positive T-SPOT result and high clinical suspicion, the patient commenced a standard anti-tuberculosis regimen (Isoniazid, Rifampicin, Pyrazinamide, and Ethambutol) postoperatively. No recurrence was observed at the 24-month follow-up.
- **Case #3 (RF Positive/JIA):** This 2-year-old patient showed initial improvement but developed recurrent mild swelling at 12 months. MRI confirmed synovial thickening and recurrence of rice bodies. The diagnosis was refined to JIA (polyarticular, RF-positive, ANA-positive). This recurrence was attributed to a failure of systemic inflammatory control rather than incomplete surgical clearance, as the patient was initially managed solely with NSAIDs postoperatively prior to the definitive JIA classification. Upon recurrence, the patient was escalated to a combination of standard DMARDs (Methotrexate) and a biologic agent (Adalimumab). At the 24-month follow-up, disease activity was controlled without further exacerbation.

### Part II: systematic literature review findings (2006–2026)

Our systematic search identified 12 relevant studies (12–23) comprising case reports and series specifically focusing on pediatric RBS (summarized in Table 3). Based on the Joanna Briggs Institute (JBI) critical appraisal tools, all included studies demonstrated acceptable methodological quality with low to moderate risk of bias, rendering them suitable for qualitative synthesis.

**Table 3:** Systematic literature review: summary of 12 studies on pediatric rice body synovitis (2,006–2026).

**Author (Year)**	**Study Design**	**Patient Demographics/Site**	**Diagnosis**	**Treatment**	**Clinical Outcome**
Wang et al. (12)	Case Series (3)	6y F, 7y M, 2y F (Knees)	Synovial Chondromatosis; TB; JIA	Arthroscopy + etiologic drugs (anti-TB for T-SPOT+)	No recurrence.
Tang et al. (13)	Case Report	Child (age unspecified) (Multiple)	Polyarticular JIA	Surgery + medical therapy	Symptoms improved.
Sener et al. (14)	Retrospective (24)	Median 6.1y (Knee/Hip/Ankle)	JIA (95.8%)	Medical therapy alone or with surgery	Resolved rice bodies in some cases; emphasized RBS as JIA marker.
Faller et al. (15)	Case Report	10y F (Knee)	JIA Flare	Synovectomy + MTX (after refractory steroids)	Resolution.
d'Aleo et al. (16)	Case Report	2-month-old (Knee)	Candida septic arthritis	Amphotericin B	Full recovery.
Okura et al. (17)	Case Report	2y F (Knee)	JIA	Surgical resection	Good prognosis.
Druschel et al. (18)	Case Series (2)	<3y (Knees)	JIA	Arthroscopic synovectomy	Symptom relief.
Teramoto et al. (19)	Case Report	12y F (Knee)	JIA (Recurrent)	Synovectomy + MTX	Recurred 10 years post-initial surgery, resolved with therapy.
Cox et al. (20)	Clinical Image	Child (Knee)	JIA	Not explicitly defined	Highlighting MRI features.
Iyengar et al. (21)	Case Report	Adolescent (Wrist)	Seronegative RA	Tenosynovectomy	Good recovery.
DiVito et al. (22)	Case Report	Child (Knee)	JIA	Surgical intervention	Confirmed diagnostic value of MRI.
Cuomo et al. (23)	Case Report	4y M (Biceps sheath)	Systemic JIA	Open biopsy/excision	Rare extra-articular location managed surgically.

**Figure 5:**
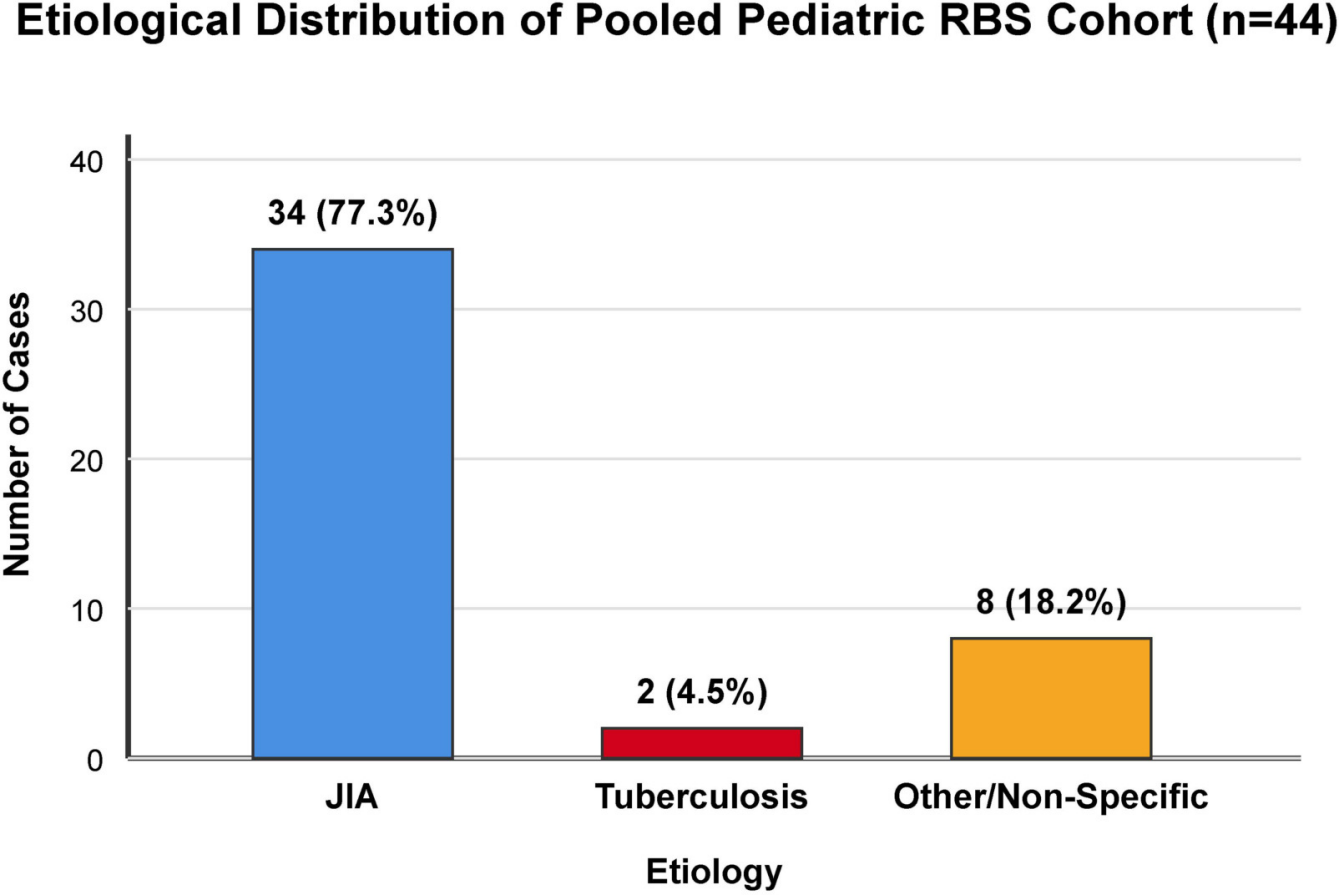
Descriptive statistical synthesis of the pooled pediatric RBS cohort (*n* = 44). The bar chart illustrates the prominent etiological shift, with Juvenile Idiopathic Arthritis (JIA) accounting for the vast majority of cases (77.3%), dwarfing historically assumed infectious origins like Tuberculosis (4.5%).

To provide a clearer and more structured synthesis from a statistical perspective, we integrated our 6 institutional cases with the 38 pediatric cases extracted from the literature, yielding a pooled analysis cohort of 44 patients (Figure 5). The descriptive analysis of this pooled cohort highlights a stark distribution in etiology. Juvenile Idiopathic Arthritis (JIA) emerged as the overwhelming predominant cause, accounting for 77.3% (*n* = 34) of all documented pediatric RBS cases globally. Infectious causes, historically the hallmark of RBS, constituted a minority, with Tuberculosis accounting for only 4.5% (*n* = 2), and fungal infection (Candida) noted in a single infant (2.3%). Non-specific or other etiologies (including synovial chondromatosis and seronegative arthritis) made up the remaining 15.9% (*n* = 7). Regarding treatment modalities, 88% of patients in the pooled cohort required surgical intervention (arthroscopy or open synovectomy) for mechanical clearance, while nearly all JIA and TB patients mandated systemic pharmacological maintenance (DMARDs or anti-TB regimens, respectively) to achieve sustained clinical remission. This robust data synthesis underscores that while pediatric RBS demands mechanical debridement for symptom relief, its contemporary identity is deeply rooted in autoimmune pathogenesis, dictating the need for targeted medical therapy.

## Discussion

Rice body synovitis in the pediatric population is an enigmatic clinical entity that serves as a sentinel for severe underlying chronic inflammation. By integrating our six-case series with a systematic review of the literature from the past two decades, this study provides a comprehensive update on the changing landscape of pediatric RBS.

### Evolving etiology: from tuberculosis to autoimmunity

Historically, the presence of rice bodies was considered pathognomonic for tuberculosis (2). Our systematic review, however, suggests an increasingly recognized association with autoimmune pathogenesis. Contemporary data, including the large cohort study by Sener et al. (14), demonstrate that Juvenile Idiopathic Arthritis (JIA) is now the predominant etiology in children, accounting for the vast majority of cases in non-endemic TB regions. This aligns with our finding in Case #3 (RF positive), where RBS was the harbinger of JIA.

Nevertheless, infectious causes cannot be entirely discounted. Case #1 in our series highlights the diagnostic dilemma posed by “non-specific” pathology in the presence of immunological evidence of TB (positive T-SPOT). As emphasized by Wang et al. (12), in regions with a high TB burden, a positive T-SPOT test in a child with granulomatous-like synovitis warrants empirical anti-tuberculous therapy to prevent the disastrous consequences of missed joint tuberculosis, even if acid-fast bacilli are not visualized. Thus, pediatric RBS should be viewed as a “diagnostic chameleon,” necessitating a broad differential that includes JIA, tuberculosis, fungal infections (especially in infants) (16), and synovial chondromatosis. Therefore, the etiological distribution must always be evaluated through the lens of local epidemiological data.

### Diagnostic specificity of MRI

While ultrasound is a useful screening tool, often showing the “sandstorm sign” with dynamic compression, MRI remains the gold standard for non-invasive diagnosis. The characteristic appearance of rice bodies—iso-to-hypointense nodules on T1WI and distinct hypointense filling defects within hyperintense effusion on T2WI—is highly specific (22). Some authors describe a “bullseye sign” or “target sign” on T2WI, representing a central core of loosely arranged fibrin surrounded by a denser collagenous rim. Clinically and radiologically, distinguishing RBS from primary synovial chondromatosis and Pigmented Villonodular Synovitis (PVNS) is crucial. Unlike synovial chondromatosis, which frequently demonstrates stippled chondroid calcifications on plain radiographs and lobulated high-signal loose bodies on MRI, rice bodies are non-calcified and appear hypointense on T2WI. Furthermore, RBS lacks the characteristic blooming artifact seen on gradient-echo (GRE) MRI sequences typical of hemosiderin deposition in PVNS (24, 25). Early recognition of these MRI patterns can expedite the surgical decision-making process.

### Therapeutic strategy: the “dual-track” approach

The management of pediatric RBS requires a “dual-track” strategy combining surgical intervention with medical management.

**Figure 6:**
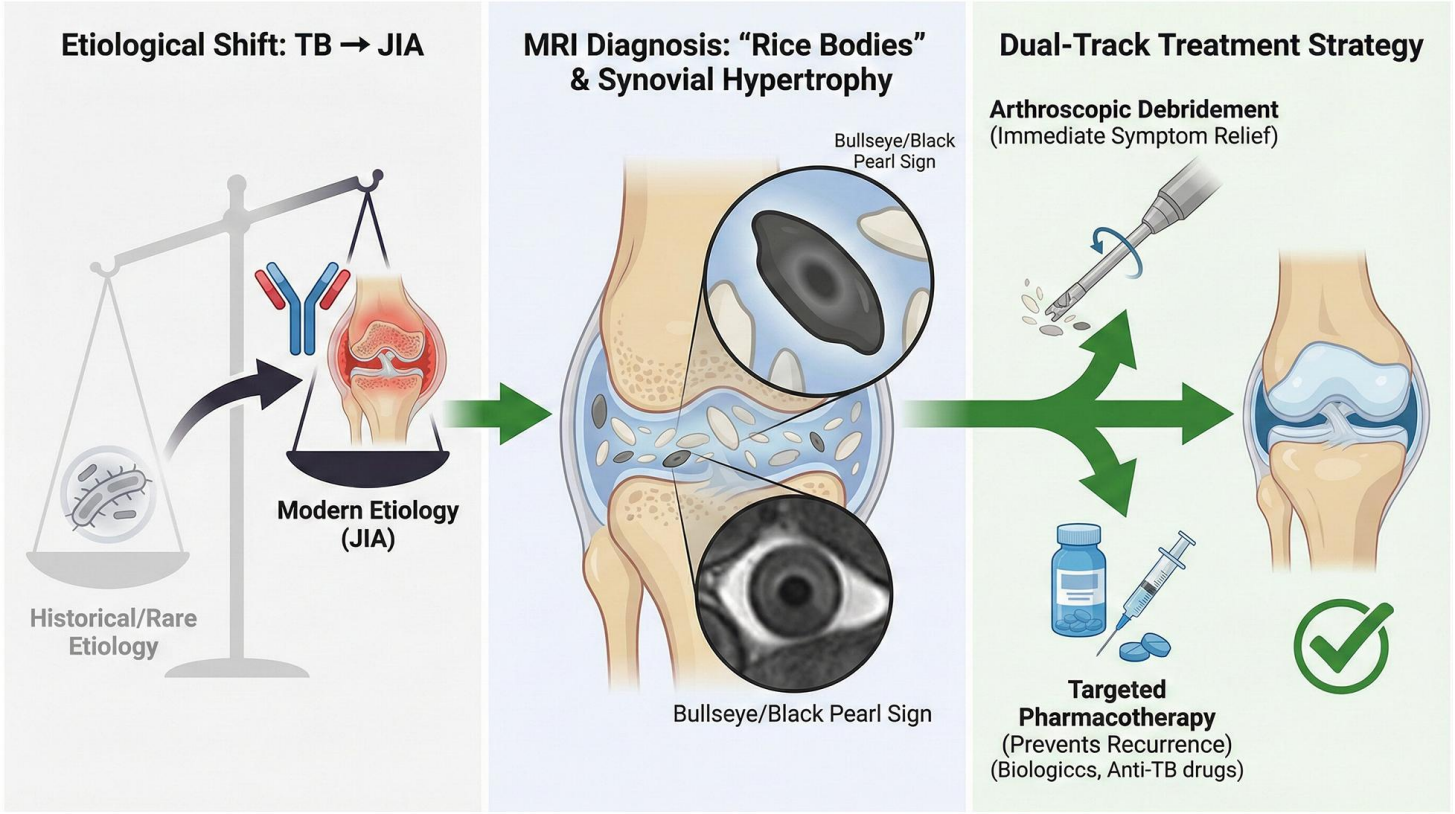
Proposed management paradigm for pediatric rice body synovitis. The schematic summarizes the key findings of this study: (Left) The distinct etiological shift in the pediatric population from tuberculosis (historical) to Juvenile Idiopathic Arthritis (modern); (Center) The characteristic “black pearl” or “bullseye” sign on T2-weighted MRI which is pivotal for early diagnosis; and (Right) The “dual-track” therapeutic strategy advocated in this study. This approach combines arthroscopic debridement for immediate symptom relief with targeted pharmacotherapy (biologics, DMARDs, or anti-TB drugs) to effectively prevent recurrence.

1. Surgical Clearance: Arthroscopic debridement is the cornerstone of immediate symptom relief. It mechanically removes the high burden of fibrin bodies and the hypertrophic synovial “factory” producing them. Our results, showing dramatic improvements in Lysholm scores, validate the efficacy of arthroscopy over open surgery, offering faster recovery and better cosmetic outcomes for children.
2. Medical Maintenance: Surgery alone is often insufficient for long-term control, especially in JIA-associated RBS. The recurrence in Case #3 at 12 months underscores this reality. Literature suggests that rice bodies can reform if the underlying inflammatory driver is not suppressed (15). Therefore, postoperative initiation of DMARDs (e.g., methotrexate, biologics) for JIA or a full course of anti-tuberculous chemotherapy for TB is mandatory. Based on the integration of our case series and the systematic review, we propose a standardized management paradigm (Figure 6). This “dual-track” strategy illustrates that while arthroscopy provides mechanical clearance, the concurrent use of etiology-specific pharmacotherapy is the linchpin for preventing recurrence. The concept that RBS is a marker of high disease activity or a “flare” in JIA (15) suggests that these patients may require more aggressive initial medical therapy.

### Limitations

This study has several limitations. First, despite being a case series, the sample size is small due to the rarity of the condition. Second, the follow-up period (mean 18.5 months) is relatively short; given that JIA recurrences can occur years later (19), longer surveillance is required. Third, genetic testing for susceptibility to autoimmune diseases was not routinely performed. Furthermore, the systematic review component is inherently susceptible to publication bias, as atypical presentations or cases with highly successful outcomes are disproportionately more likely to be published in case reports. Selection bias is also a factor, given that our institutional cohort and the majority of the literature reports originate from tertiary referral centers treating more refractory cases (26).

## Conclusions

Pediatric Rice Body Synovitis is a rare but distinct indicator of chronic joint inflammation, showing an emerging trend as being most commonly associated with JIA in the modern era, though tuberculosis remains a critical differential. MRI is the diagnostic modality of choice. Effective management relies on a multidisciplinary approach: prompt arthroscopic debridement to preserve joint function and rigorous, etiology-specific pharmacological treatment (anti-rheumatic or anti-tuberculous) to prevent recurrence. Critically, the interpretation of etiology must be contextualized by geographical variability; while JIA predominates in developed nations, mycobacterial infections remain a paramount diagnostic priority in tuberculosis-endemic regions. Future research should focus on the long-term prognostic implications of RBS in JIA patients.

## Data Availability

The original contributions presented in the study are included in the article/Supplementary Material, further inquiries can be directed to the corresponding author.

## References

[1] Gillijns M Vandesande W. Rice bodies in the wrist. Mod Rheumatol Case Rep. (2022) 6(1):150–4. 10.1093/mrcr/rxab040 34614514

[2] Guo JJ Wu K Xu Y Yang H. Hundreds of rice bodies in the subacromial-subdeltoid Bursa: report of two cases and literature review. BMC Musculoskelet Disord. (2020) 21(1):539. 10.1186/s12891-020-03563-0 32787818 PMC7424980

[3] Lin Z Zhang X Chen C Xu Y. Rice body due to lupus. Korean J Intern Med. (2023) 38(6):947–8. 10.3904/kjim.2023.103 37334509 PMC10636552

[4] Fujieda Y Ninagawa K Matsui Y Kono M Kamishima T Iwasaki N Non-tuberculosis mycobacterium tenosynovitis with rice bodies in a patient with systemic lupus erythematosus. Int Med (Tokyo, Japan). (2020) 59(18):2317–20. 10.2169/internalmedicine.4671-20 PMC7578613 32536648

[5] Popert AJ Scott DL Wainwright AC Walton KW Williamson N Chapman JH. Frequency of occurrence, mode of development, and significance or rice bodies in rheumatoid joints. Ann Rheum Dis. (1982) 41(2):109–17. 10.1136/ard.41.2.109 6176192 PMC1000892

[6] Thevenon A Cocheteux P Duquesnoy B Mestdagh H Lecomte-Houcke M Delcambre B. Subacromial bursitis with rice bodies as a presenting feature of seronegative rheumatoid arthritis. Arthritis Rheum. (1987) 30(6):715–6. 10.1002/art.1780300618 3606687

[7] Tian Y Zhou HB Yi K Wang KJ. Idiopathic tenosynovitis of the wrist with multiple rice bodies: a case report and review of literature. World J Clin Cases. (2022) 10(32):11908–20. 10.12998/wjcc.v10.i32.11908 36405290 PMC9669876

[8] Ong HY Algazwi DAR Muhamat Nor FE Hallinan J. Fungal abscess mimicking ischiogluteal bursitis with rice bodies. J Clin Rheumatol. (2021) 27(1):e17–8. 10.1097/RHU.0000000000001206 31804254

[9] Kassimos D George E Kirwan JR. Rice bodies in the pleural aspirate of a patient with rheumatoid arthritis. Ann Rheum Dis. (1994) 53(6):427–8. 10.1136/ard.53.6.427 8037504 PMC1005363

[10] Mathew G Sohrabi C Franchi T Nicola M Kerwan A Agha R. Preferred reporting of case series in surgery (PROCESS) 2023 guidelines. Int J Surg (London, England). (2023) 109(12):3760–9. 10.1097/JS9.0000000000000940 PMC10720832 37988417

[11] Page MJ McKenzie JE Bossuyt PM Boutron I Hoffmann TC Mulrow CD The PRISMA 2020 statement: an updated guideline for reporting systematic reviews. BMJ (Clinical Research ed). (2021) 372:n71. 10.1136/bmj.n71 33782057 PMC8005924

[12] Wang L Jin Y Huang H Yang Z Ding F Xu X Rice body synovitis in pediatrics: three different case reports. Front Pediatr. (2024) 12:1391229. 10.3389/fped.2024.1391229 38938505 PMC11210276

[13] Tang T Tang X. Polyarticular juvenile idiopathic arthritis with rice bodies formation: a case report. Int J Rheum Dis. (2024) 27(5):e15184. 10.1111/1756-185X.15184 38798092

[14] Sener S Tanali G Ergen FB Kasap Cuceoglu M Balik Z Bayindir Y Rice bodies in children with rheumatic disorders: a case series and systematic literature review. Mod Rheumatol. (2023) 33(4):811–6. 10.1093/mr/roac075 35819010

[15] Faller G Haagensen M Barrow M. Juvenile idiopathic arthritis flare due to rice bodies in the knee of a 10-year-old girl. S Afr Med J. (2018) 108(10):833–5. 10.7196/SAMJ.2018.v108i10.13228 30421710

[16] d'Aleo F Bonanno R Midiri A Mancuso G Cordaro S Warm A A case of Candida septic arthritis with rice body formation in a 2-month-old infant. Infez Med. (2017) 25(4):374–6. https://pubmed.ncbi.nlm.nih.gov/29286020/29286020

[17] Okura Y Tsumagari S Nawate M Yoshioka M Shikano T Takahashi Y. Juvenile idiopathic arthritis with rice bodies in a 2-year-old girl. J Pediatr. (2016) 172:220. 10.1016/j.jpeds.2016.01.039 26858191

[18] Druschel C Funk JF Kallinich T Lieb A Placzek RP. Development of rice bodies in 2 children younger than 3 years. J Clin Rheumatol. (2013) 19(1):35–7. 10.1097/RHU.0b013e31826d6b5e 23319022

[19] Teramoto A Watanabe K Kii Y Kudo M Otsubo H Wada T Recurrent knee arthritis diagnosed as juvenile idiopathic arthritis with a 10-year asymptomatic period after arthroscopic synovectomy: a case report. J Med Case Rep. (2013) 7:166. 10.1186/1752-1947-7-166 23805989 PMC3700890

[20] Cox A Allen R Akikusa J. Rice bodies in juvenile idiopathic arthritis: a clinical image. J Paediatr Child Health. (2012) 48(3):280. 10.1111/j.1440-1754.2012.02420.x 22417465

[21] Iyengar K Manickavasagar T Nadkarni J Mansour P Loh W. Bilateral recurrent wrist flexor tenosynovitis and rice body formation in a patient with sero-negative rheumatoid arthritis: a case report and review of literature. Int J Surg Case Rep. (2011) 2(7):208–11. 10.1016/j.ijscr.2011.07.001 22096729 PMC3199677

[22] DiVito A Kan JH. Juvenile idiopathic arthritis with rice bodies. Pediatr Radiol. (2008) 38(11):1263. 10.1007/s00247-008-0978-7 18751974

[23] Cuomo A Pirpiris M Otsuka NY. Case report: biceps tenosynovial rice bodies. J Pediatr Orthop B. (2006) 15(6):423–5. 10.1097/01.bpb.0000228392.62678.df 17001249

[24] Singh R Goyal A Srivastava DN Gautam D. Giant melon seed bodies in painless shoulder swelling. Indian J Musculoskelet Radiol. (2021) 3(1):57–9. 10.25259/IJMSR_38_2020

[25] Qi W Ren Y Wang H Wan Y Pan H Yao J. Candida parapsilosis-caused arthritis with rice body formation: a case presentation and literature review. Infect Drug Resist. (2023) 16:4123–35. 10.2147/IDR.S416990 37396064 PMC10312336

[26] Murad MH Sultan S Haffar S Bazerbachi F. Methodological quality and synthesis of case series and case reports. BMJ Evid Based Med. (2018) 23(2):60–3. 10.1136/bmjebm-2017-110853 29420178 PMC6234235

